# Regionalization of Head and Neck Oncology Tumor Boards: Perspectives of Collaborating Physicians

**DOI:** 10.1002/oto2.18

**Published:** 2023-02-28

**Authors:** Neha B. Amin, Kelly M. Bridgham, Jessica P. Brown, Kelly F. Moyer, Rodney J. Taylor, Jeffrey S. Wolf, Matthew E. Witek, Jason K. Molitoris, Ranee Mehra, Kevin J. Cullen, John C. Papadimitriou, Prashant Raghavan, Kyle M. Hatten

**Affiliations:** ^1^ University of Maryland School of Medicine Baltimore Maryland USA; ^2^ Department of Epidemiology and Public Health University of Maryland School of Medicine Baltimore Maryland USA; ^3^ Department of Otorhinolaryngology–Head and Neck Surgery University of Maryland School of Medicine Baltimore Maryland USA; ^4^ Department of Radiation Oncology University of Maryland School of Medicine, Maryland Proton Treatment Center Baltimore Maryland USA; ^5^ Marlene and Stewart Greenebaum Comprehensive Cancer Center University of Maryland School of Medicine Maryland Baltimore USA; ^6^ Department of Pathology University of Maryland School of Medicine Baltimore Maryland USA; ^7^ Department of Diagnostic Radiology and Nuclear Medicine University of Maryland School of Medicine Baltimore Maryland USA

**Keywords:** head and neck cancer, medical oncology, multidisciplinary tumor board, radiation oncology

## Abstract

**Objectives:**

To survey academic and community physician preferences regarding the virtual multidisciplinary tumor board (MTB) for further improvement and expansion.

**Study Design:**

This anonymous 14‐question survey was sent to individuals that participated in the head and neck virtual MTBs. The survey was sent via email beginning August 3, 2021, through October 5, 2021.

**Setting:**

The University of Maryland Medical Center and regional practices in the state of Maryland.

**Methods:**

Survey responses were recorded and presented as percentages. Subset analysis was performed to obtain frequency distributions by facility and provider type.

**Results:**

There were 50 survey responses obtained with a response rate of 56%. Survey participants included 11 surgeons (22%), 19 radiation oncologists (38%), and 8 medical oncologists (16%), amongst others. More than 96% of participants found the virtual MTB to be useful when discussing complex cases and impactful to future patient care. A majority of respondents perceived a reduction in time to adjuvant care (64%). Community and academic physician responses strongly agreed that the virtual MTB improved communication (82% vs 73%), provided patient‐specific information for cancer care (82% vs 73%), and improved access to other specialties (66% vs 64%). Academic physicians, more so than community physicians, strongly agreed that the virtual MTB improves access to clinical trial enrollment (64% vs 29%) and can be useful in obtaining CME (64% vs 55%).

**Conclusion:**

Academic and community physicians view the virtual MTB favorably. This platform can be adapted regionally and further expanded to improve communication between physicians and improve multidisciplinary care for patients.

Multidisciplinary team management of head and neck cancer offers a comprehensive approach to the diagnosis and management of patient care. Team members from various disciplines collectively host a multidisciplinary tumor board (MTB), which reviews each patient's clinical presentation and provides a consensus recommendation from the group. In addition, MTBs ensure adherence to clinical practice guidelines and offer the opportunity for enrollment in clinical trials.^1^, ^2^, ^3^, ^4^ This collaboration between oncologists and various interprofessional team members positively impacts patient survival.^5^, ^6^

Early trials of MTBs, from in‐person meetings to a virtual setting, have rapidly evolved into widespread adoption since the COVID‐19 pandemic. In addition to reducing the risk of transmission of respiratory illnesses, these virtual meetings have demonstrated the ability to easily facilitate communication between physically distant providers and have been shown to increase case presentations.^4^, ^7^, ^8^ A 6‐month survey of participants in the lung cancer virtual MTB found that 95% believed the virtual MTB to be feasible and acceptable as well as impactful with regards to optimizing the flow of clinical information (91%), equity of care (96%) and specialist collaboration (88%).^9^ Virtual MTBs have been adapted by various specialties evaluating lung cancer, breast cancer, gastrointestinal cancers, lymphoma, and head, and neck cancer.^8^, ^9^, ^10^, ^11^, ^12^, ^13^ The virtual MTB plays an important role in changing management. A study of the multidisciplinary lymphoma virtual tumor board demonstrated changes in radiologic interpretation (1.9%), pathologic diagnosis (9.4%), and clinical management (30%).^11^ Virtual programs have been shown to improve quality, adherence, and timelines of multidisciplinary care while reducing travel burdens.^12^ Despite some limitations that include technical difficulties and technological costs, virtual tumor boards have been proven to be proficient in facilitating cancer care.^8^, ^14^, ^15^

Virtual MTBs have become a widely adopted means of communicating within a multidisciplinary system. The platform offers an opportunity to create an accessible program that can be expanded to accommodate an inter‐institutional regional cancer network.^8^ While virtual MTBs have been associated with quality oncologic care, the emerging collaboration and priorities between community and academic physicians are not well studied and should be evaluated to further improve this developing platform. The objective of this study is to survey provider preferences and perceived value of head and neck cancer virtual MTBs in order to define priorities for future MTB collaborations.

## Methods

This study was evaluated by the University of Maryland, Baltimore, Institutional Review Board (IRB) and was granted IRB exemption based on the anonymous nature of the survey data. A survey was provided to oncology providers participating in the virtual MTB hosted by the University of Maryland Medical Center (UMMC). Survey topics were determined based on a review of the literature as well as the tumor board experience at UMMC. The survey was designed in collaboration with the study coauthor and member of the Department of Epidemiology and Public Health of the University of Maryland School of Medicine.

**Figure 1:**
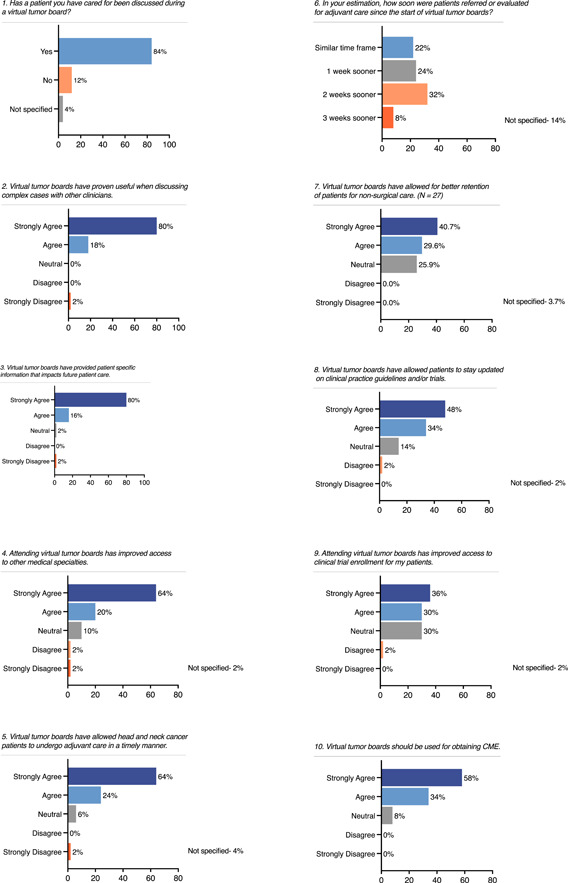
Survey responses.

**Figure 2:**
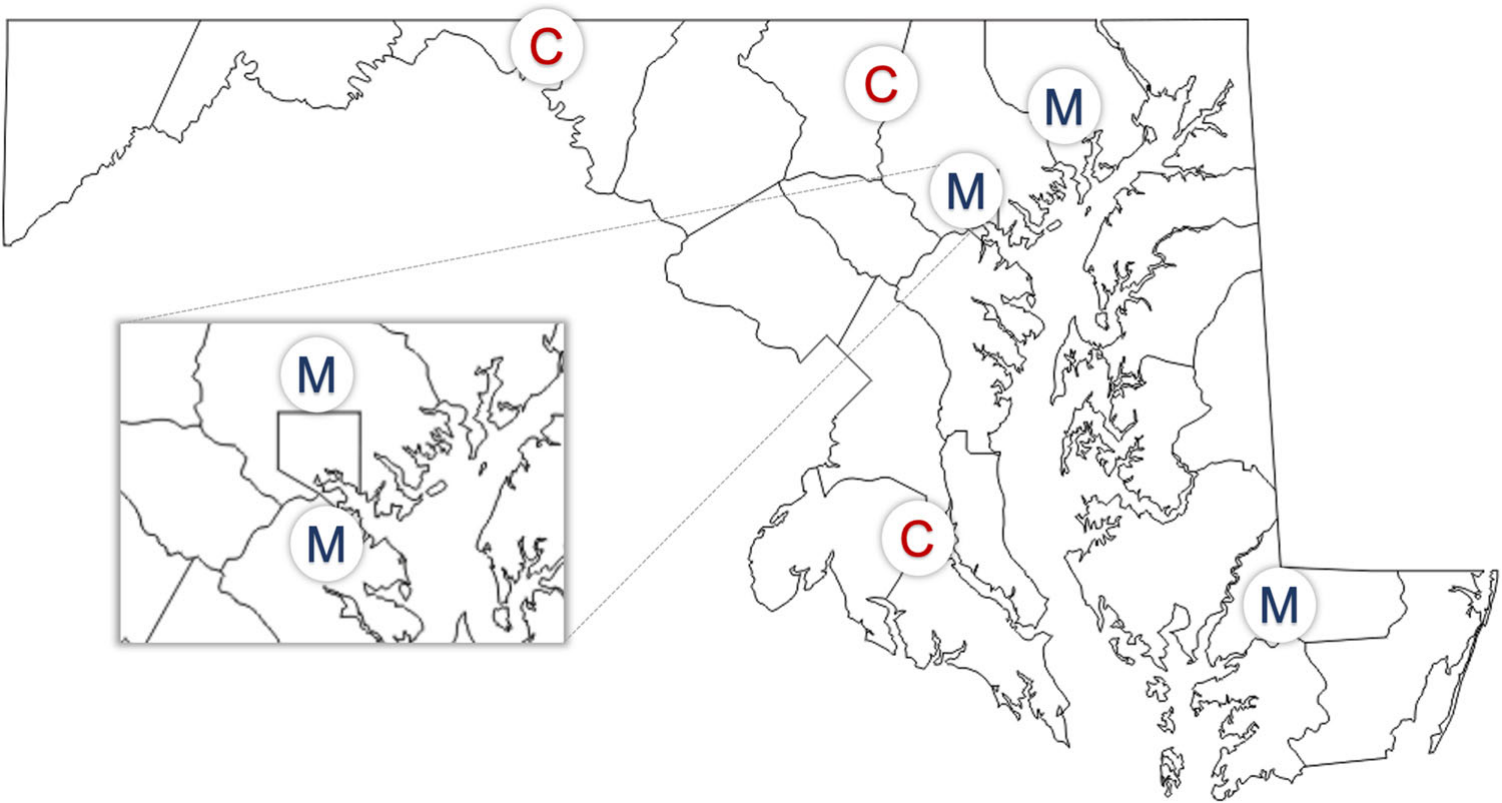
Map of University of Maryland Medical Center multidisciplinary tumor board regional network. C, Community Center; M, University of Maryland Medical System. Map template obtained from https://d-maps.com/carte.php?num_car=20591&lang=en.

A 14‐question survey was distributed to members participating in an interinstitutional tumor board (Table 1 and Figure 1). The responses to the survey were based on the experiences of the MTBs initiated in June 2020 due to the COVID‐19 pandemic. Inter‐institutional meetings are held regularly and are composed of a variety of specialists, including otolaryngologists, neuroradiologists, radiation, and medical oncologists, pathologists, and other cancer care coordinators. Survey responses from tumor board participants represent convenience sampling, which includes a nonprobability sampling method of participants who were likely closely involved in tumor board meetings. Providers are located among a network of hospitals in Maryland, including the University of Maryland Medical System, as well as three independent regional cancer centers (Figure 2). Case presentations are organized and led by an attending surgeon and include a multidisciplinary team to review patient history, pathology, and radiology details. The case is then discussed among the oncologists to determine the optimal treatment regimen. Between June 2020 and December 2021, 123 patients were presented using this virtual platform.

**Table 1:** Survey Respondent Demographics

Total responses	50
Gender	
Male	23 (46.0%)
Female	23 (46.0%)
Unknown	4 (8.0%)
Years in practice	
<1 year	1 (2.0%)
1‐5 years	10 (20.0%)
6‐10 years	9 (18.0%)
11‐20 years	13 (26.0%)
>20 years	17 (34.0%)
Cancer care service	
Surgeon	11 (22.0%)
Radiation oncologist	19 (38.0%)
Medical oncologist	8 (16.0%)
Pathologist	2 (4.0%)
Neuroradiologist	2 (4.0%)
Other	8 (16.0%)
Practice location	
Main medical center	11 (22.0%)
Out of system regional partner	25 (50.0%)
In system medical center	13 (26.0%)
Not specified	1 (2.0%)

The survey was developed to assess the opinions of head and neck tumor board attendees. The anonymous survey was distributed via a tumor board email list serve and established in the Research Electronic Data Capture (REDCap) platform hosted at the University of Maryland School of Medicine. Responses were collected from August 3, 2021, to October 5, 2021. Respondents included head and neck surgeons, radiation oncologists, medical oncologists, neuroradiologists, and other attendees of the UMMC and regional tumor board network. Data obtained include demographic variables such as gender, years in practice, specialization, and practice facility type. Subsequent survey questions assessed physician agreement with various statements regarding virtual tumor boards to better understand physician preferences and priorities using this platform. Subset analysis using Pearson's *χ* ^2^ test was performed to evaluate any differences in survey responses based on cancer care specialty or practice location and to obtain frequency distributions by facility and provider type. All analysis was conducted using IBM SPSS Statistics for Macintosh, Version 27.0., IBM Corp.

## Results

In total, 50 survey responses were obtained out of 89 recipients. The response rate was 56%. Survey participants included 11 surgeons (22%), 19 radiation oncologists (38%), and 8 medical oncologists (16%), with the remaining participants being pathologists, neuroradiologists, or other cancer care coordinators. Thirty‐eight respondents (76%) practiced at community cancer centers, with the remaining 11 (22%) practicing at an academic medical center. Seventeen (34%) respondents had been in practice for greater than 20 years (Table 1).

Greater than 96% of participants found the virtual MTB to be useful when discussing complex cases and impactful to future patient care. 84% and 66% found the virtual MTB to improve access to other medical specialties and clinical trial enrollment for patients, respectively. Thirty‐two (64%) respondents perceived that the virtual MTB decreased the time interval to adjuvant care. Of the 27 radiation and medical oncologists, 19 (70%) agreed that the virtual MTB allowed for better retention of patients within their practice. 46 (92%) of respondents believed that the virtual MTB should be used for obtaining Continuing Medical Education (CME) credit (Figure 1).

**Figure 3:**
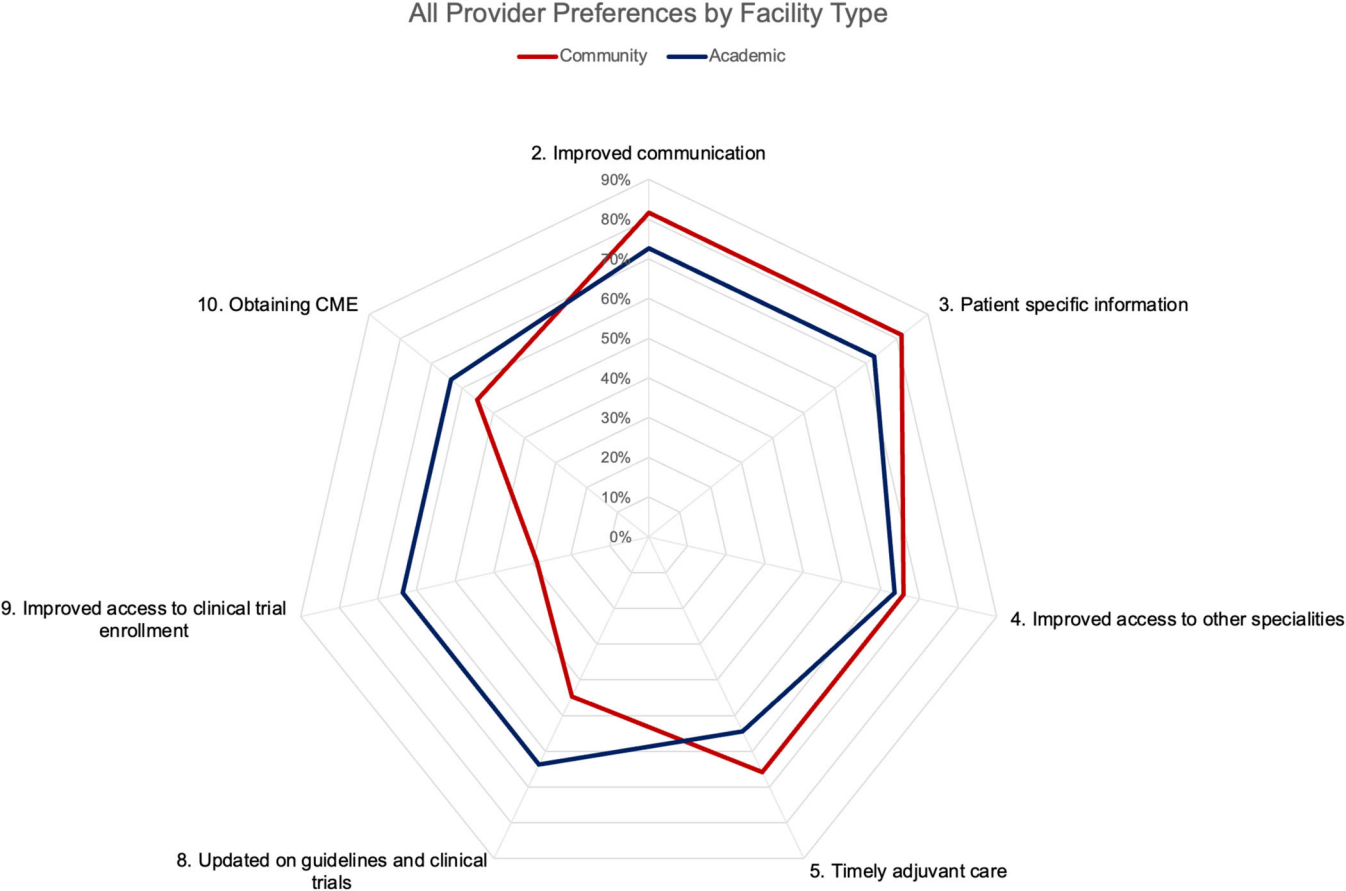
The percentages of community versus academic providers that strongly agreed with the 7 representative survey statements. Axis, % strongly agree.

Surgeon and oncologist preferences were generally similar across all MTB survey statements. Radiation and medical oncologists, more so than surgeons, strongly agreed that virtual MTBs improved communication (85% vs 64%), provided patient‐specific information useful to cancer care (85% vs 55%), and improved access to other specialties (63% vs 55%). Figure 3 represents the percentage of participants that strongly agreed with MTB survey statements based on their practice location. A larger percentage of academic physicians versus community physicians strongly agreed that the virtual MTB improves access to clinical trial enrollment (64% vs 29%) and can be useful in obtaining CME (64% vs 55%).

**Figure 4:**
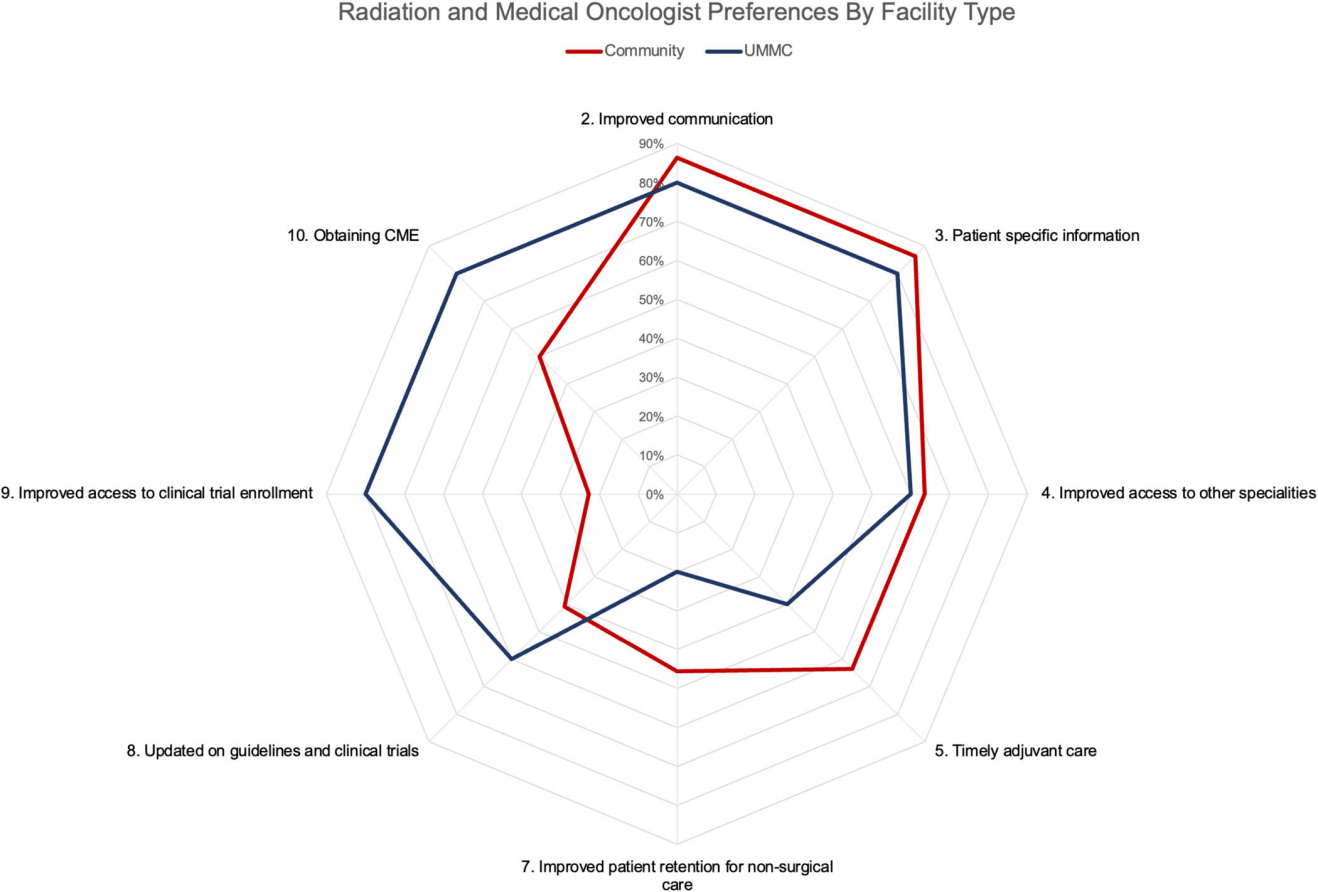
The percentages of community versus academic oncologists that strongly agreed with the 8 representative survey statements. Axis, % strongly agree.

Survey responses, specifically among medical and radiation oncologists located at academic centers compared to those at community centers, are represented in Figure 4. A greater proportion of community oncologists noted that the virtual MTB offers timely adjuvant therapy compared to academic oncologists (64% vs 40%). Responses to the oncologist‐specific question regarding the retention of nonsurgical patients differed between community and academic oncologists. 46% of community oncologists responded that the virtual MTB allowed for better retention of nonsurgical patients compared to 20% of academic oncologists. Academic oncologists, more so than community oncologists, agreed that virtual MTBs have allowed patients to stay up to date on clinical practice guidelines (60% vs 41%), improved clinical trial access (80% vs 23%), and should qualify for obtaining CME credit (80% vs 50%).

Subset analysis did not demonstrate significant differences between responses across specialty or facility types.

## Discussion

The virtual MTB provides an effective means for the multidisciplinary management of head and neck cancer patients. The survey results demonstrate that this platform is viewed favorably by cancer care providers, regardless of specialty or practice location. Respondents indicated that virtual MTBs improved the patient care experience at several crucial points, including improved communication and decreased time to adjuvant therapy. Communication between physically distant community centers offered value in reviewing patient‐specific information with increased access to various providers. In addition, time to adjuvant radiation was perceived to have been reduced with improved postoperative communication.

Generally, all providers, including surgeons, radiation oncologists, and medical oncologists, strongly agreed that the virtual MTB platform demonstrated improvements in communication and timely and evidence‐based care, as well as increased access to clinical trials. It is notable that survey responses differed between oncologists in a community setting versus an academic institution. A larger proportion of community radiation and medical oncologists found that virtual MTBs improved the time to initiation of adjuvant treatment. Similar to academic oncologists, the majority of community oncologists also strongly agreed that virtual MTBs were impactful to patient care while also increasing access to other medical specialties. This demonstrates that virtual MTBs can be particularly useful in community‐based cancer centers, where a virtual platform may increase access to other disciplines, such as surgical oncologists at academic institutions. Close to half of all community radiation and medical oncologists also agreed that virtual MTBs improved patient retention for nonsurgical care, specifically patients that prefer seeking nonsurgical care closer to home versus at an academic medical center. In this setting, virtual MTBs can allow academic oncologists to remain informed about patient chemoradiation treatment outside the institution.

Academic oncologists, more so than community providers, agreed that virtual MTBs improved access to clinical trial enrollment. The virtual MTB can be used to spread awareness of clinical trials regionally to provide recurrent/metastatic cancer patients with more options for treatment. In addition, this model can potentially improve clinical trial access and enrollment for academic centers. However, due to this virtual MTB's implementation during the COVID‐19 pandemic, institutional restrictions on research hindered more robust clinical trial enrollment.

Most respondents acknowledged that the virtual MTB has allowed patients to undergo adjuvant care in a shorter time period. Efficiency in the transition of care from definitive surgery to adjuvant therapy is associated with improved tumor control and survival.^16^, ^17^ Increases in treatment delays are attributed to the growing complexity of head and neck cancer management.^18^ In addition, patients who receive adjuvant radiation therapy at a different facility have been found to have greater treatment delays.^19^ These treatment delays are potentially associated with inefficiencies of transfer of care that may be addressed by the virtual MTB. Coordinated multidisciplinary treatment teams are well adopted within institutional practices and have identified improvements in time to adjuvant treatment. Kelly et al^2^ noted patients managed with the multidisciplinary approach received adjuvant radiation earlier (48.2 vs 60.9 days, *p* = 0.009).

Despite this study providing the largest survey report of a multidisciplinary tumor board, this study is not without limitations. This study's survey has not been validated in part due to the absence of gold‐standard questionaries that measure tumor board efficacy. Additionally, survey respondents consisted of providers within the state of Maryland, potentially limiting the generalizability of our results. Furthermore, our results are subject to nonresponse bias, given the relatively high nonresponse rate of 44% in this study. Another aspect of this study was to understand physician opinions and perceptions of timely adjuvant care, however, this is a limitation as the reduction of time to adjuvant care was not precisely measured. Nonetheless, this study is the largest survey of physician opinions regarding virtual MTBs over an 18‐month period and contributes to the growing body of literature supporting this platform.

The virtual MTB experience has been viewed favorably and is amenable to further implementation and expansion. While head and neck cancer tumor boards are traditionally hosted at a single institution, inter‐institutional programs improve access to medical specialties. This novel program provides streamlined predefined steps to coordinate care, from clinic visits to obtaining imaging and laboratory tests and finally to treatment initiation.^20^

## Conclusion

Virtual tumor boards are an effective platform for the multidisciplinary management of head and neck cancer patients across high‐volume academic and community‐based cancer centers. Overall, providers across all specialties and locations view the virtual MTB favorably, with an improvement in care communication and coordination. The virtual MTB may improve access to other medical specialties, particularly for providers based in the community and/or regional locations. We recommend the continued utilization of the virtual MTB beyond the COVID‐19 pandemic to facilitate the use of multimodal care across a regional network.

## Author Contributions

**Neha B. Amin**, survey design, analysis, manuscript drafting; **Kelly M. Bridgham**, survey design, analysis, manuscript drafting; **Jessica P. Brown**, survey design, analysis, manuscript review; **Kelly F. Moyer**, study design, manuscript review; **Rodney J. Taylor**, study design, manuscript review; **Jeffrey S. Wolf**, study design, manuscript review; **Matthew E. Witek**, study design, manuscript review; **Jason K. Molitoris**, study design, manuscript review; **Ranee Mehra**, study design, manuscript review; **Kevin J. Cullen**, study design, manuscript review; **Prashant Raghavan**, study design, manuscript review; **Kyle M. Hatten**, study design, analysis, manuscript drafting, and review.

## Disclosures

### Competing interests

The authors declare that there are no conflicts of interest.

### Sponsorships

None.

### Funding source

None.
